# Mitochondria Isolated from Hearts Subjected to Ischemia/Reperfusion Benefit from Adenine Nucleotide Translocase 1 Overexpression

**DOI:** 10.3390/membranes11110836

**Published:** 2021-10-29

**Authors:** Andrea Dörner, Oleg Lynetskiy, Gerhild Euler, Ulf Landmesser, Klaus-Dieter Schlüter, Jacqueline Heger

**Affiliations:** 1 Department of Cardiology, Campus Benjamin Franklin, Charité–Universitätsmedizin Berlin, Corporate Member of Freie Universität Berlin, Humboldt-Universität zu Berlin and Berlin Institute of Health, 12200 Berlin, Germany; andrea.doerner@charite.de (A.D.); Ulf.Landmesser@charite.de (U.L.); 2 DZHK, German Centre for Cardiovascular Research, Partner Site Berlin, 13347 Berlin, Germany; 3 Physiologisches Institut, Justus-Liebig-Universität Gießen, 35392 Gießen, Germany; Oleg-Lynetskiy@gmx.de (O.L.); Gerhild.Euler@physiologie.med.uni-giessen.de (G.E.); Klaus-Dieter.Schlueter@physiologie.med.uni-giessen.de (K.-D.S.)

**Keywords:** adenine nucleotide translocase 1 (ANT1), Langendorff-perfused hearts, mitochondria, ischemia/reperfusion (I/R)

## Abstract

Reperfusion is the only feasible therapy following myocardial infarction, but reperfusion has been shown to damage mitochondrial function and disrupt energy production in the heart. Adenine nucleotide translocase 1 (ANT1) facilitates the transfer of ADP/ATP across the inner mitochondrial membrane; therefore, we tested whether ANT1 exerts protective effects on mitochondrial function during ischemia/reperfusion (I/R). The hearts of wild-type (WT) and transgenic ANT1-overexpressing (ANT1-TG) rats were exposed to I/R injury using the standard Langendorff technique, after which mitochondrial function, hemodynamic parameters, infarct size, and components of the contractile apparatus were determined. ANT1-TG hearts expressed higher ANT protein levels, with reduced levels of oxidative 4-hydroxynonenal ANT modifications following I/R. ANT1-TG mitochondria isolated from I/R hearts displayed stable calcium retention capacity (CRC) and improved membrane potential stability compared with WT mitochondria. Mitochondria isolated from ANT1-TG hearts experienced less restricted oxygen consumption than WT mitochondria after I/R. Left ventricular diastolic pressure (Pdia) decreased in ANT1-TG hearts compared with WT hearts following I/R. Preserved diastolic function was accompanied by a decrease in the phospho-lamban (PLB)/sarcoplasmic reticulum calcium ATPase (SERCA2a) ratio in ANT1-TG hearts compared with that in WT hearts. In addition, the phosphorylated (P)-PLB/PLB ratio increased in ANT1-TG hearts after I/R but not in WT hearts, which indicated more effective calcium uptake into the sarcoplasmic reticulum in ANT1-TG hearts. In conclusion, ANT1-TG rat hearts coped more efficiently with I/R than WT rat hearts, which was reflected by preserved mitochondrial energy balance, diastolic function, and calcium dynamics after reperfusion.

## 1. Introduction

Myocardial infarction (MI) causes blood vessel occlusion, interrupting the delivery of oxygen and substrates to the heart. This process impairs mitochondrial ATP production and harms the permeability barrier of the inner mitochondrial membrane [1]. Reperfusion, which is an essential component of MI therapy, restores the supply of oxygen and substrates to the heart; however, reperfusion also exerts additional mechanical and bioelectrical stress to the myocardium [2]. Reperfusion exacerbates the opening of the mitochondrial permeability transition pore (MPTP), further impairing mitochondrial function [3]. In addition, reperfusion leads to an increase in end-diastolic pressure [4] due to changes in intracellular Ca^2+^ dynamics, associated with the downregulation of the sarcoplasmic reticulum (SR) Ca^2+^-ATPase 2 (SERCA2) [5], which deteriorates heart function and the onset of heart failure (HF).

Adenine nucleotide translocase (ANT) is a highly expressed transmembrane protein located in the inner mitochondrial membrane. ANT acts as an ADP/ATP carrier, exchanging intra-mitochondrial ATP^4−^ with extra-mitochondrial ADP^3−^ [6,7], and plays key physiological roles in cellular metabolism. In addition, ANT modulates MPTP, thereby influencing the mitochondrial membrane potential [8,9]. ANT1 is the predominant ANT isoform expressed in the heart, and ANT1 mutations and dysfunction have been associated with mitochondrial stress, respiratory chain dysfunction, mitochondrial DNA (mtDNA) instability, and severe cardiac and muscular disorders [10,11]. In contrast, increased ANT1 expression or activity have been found to have cell-protective effects that affect energy metabolism, intra- and intercellular communications, and contractile function [12,13,14].

We recently demonstrated that ANT1 has protective effects in chronic ischemic hearts [15]. However, from a medical perspective, mechanisms must be identified that protect cardiac processes during reperfusion, which represents the primary and essential therapeutic intervention following MI. Reperfusion is especially harmful to cardiac mitochondria, resulting in spontaneous over-oxygenation and the massive opening of the MPTP. Consequently, we investigated whether ANT1 overexpression protects against I/R injury by analyzing hemodynamic parameters, infarct size, mitochondrial function, and expression of calcium-handling proteins in ANT1-TG hearts subjected to I/R injury. 

## 2. Materials and Methods

### 2.1. Animals

All animals were maintained under conditions that conform to the *Guide for the Care and Use of Laboratory Animals*, published by the US National Institutes of Health (NIH Publication No. 85–23, revised 1996). This study was approved by the institutional animal care committee of the Justus-Liebig-University Giessen and was registered under the number 469_M. ANT1-overexpressing rats have previously been characterized in detail by Walther et al. (2007) [12].

### 2.2. Langendorff Perfusion (Volume-Constant Mode)

ANT1-TG[RjHAN:SD-Tg(Myh6-SLC25A4)^FEM^ (previously; TGRMHCrANT1[ANT])] and wild-type (WT) Sprague Dawley rats [RjHAN:SD] (4- to 6-month-old) were sedated with isoflurane and sacrificed by cervical dislocation. To connect hearts to the Langendorff apparatus, the hearts were quickly excised, and the aorta was cannulated and retrogradely perfused with a 16-gauge needle. A plastic balloon was inserted into the left ventricle through the mitral valve and connected to a pressure transducer to continuously measure left ventricular (LV) pressure. A second transducer measured coronary perfusion pressure. The hearts were perfused with an oxygenated buffer (pH 7.4, 37 °C) containing 140 mmol/L NaCl, 24 mmol/L NaHCO_3_, 2.7 mmol/L KCl, 0.4 mmol/L KH_2_PO_4_, 1 mmol/L MgSO_4_, 1.8 mmol/L CaCl_2_, and 5 mmol/L glucose, which was maintained at 95% O_2_ and 5% CO_2_. The hearts were allowed to stabilize for 20 min. The inflated balloon maintained a diastolic pressure of 10 mmHg. The flow rate was adjusted to a subsequent perfusion pressure of 50 mmHg and was maintained constant after that. After setting the specified parameters and reaching a stable stage, a stabilization phase of a further ten minutes followed. For I/R experiments, the hearts were then subjected to 45 min of global ischemia, followed by two hours of reperfusion. For normoxic experiments, the hearts were subjected to 15 min of normoxia after a stabilization period. Previous studies have demonstrated that this is an appropriate control [16], as discussed in more detail in the discussion part. The maximal rate of left ventricular pressure (dP/dt_max_), the minimal rate of left ventricular pressure (dP/dt_min_), systolic pressure (P_sys_), and diastolic pressure (P_dia_) were measured. All parameters were continuously recorded (8 channels, MacLab AD Instruments, Paris, France) and were measured before and at the end of the stabilization period and at various time points during I/R [17].

### 2.3. Infarct Size Measurement

After 45 min of ischemia and two hours of reperfusion, whole hearts were rapidly removed, sliced (9 to 10 slices per heart, starting from the apex of the heart), and directly incubated in 2, 3, 5-triphenyl tetrazolium chloride (TTC) solution (1%) for ten minutes. The slices were then fixed in a buffered 10% formalin solution for 24 h before being photographed. The infarct size in each heart was quantified by a blinded observer, using a computerized planimetric technique (ImageJ software, NIH, Bethesda, MD, USA). The infarct size was expressed as a percentage of the left ventricle (LV) size [18].

### 2.4. Real-Time RT-PCR Analysis

Total RNA from cardiomyocytes was extracted with Trizol (Invitrogen, Karlsruhe, Germany), according to the manufacturer’s instructions. cDNA was synthesized using a QuantiTect Reverse Transcription Kit (Qiagen, # 205313 with DNA digestion). Aliquots (1.5 µL) of synthesized cDNA were used to perform real-time reverse transcriptase-polymerase chain reaction (RT-PCR), in a final volume of 10 µL, containing primer pairs at 1.5 µmol/L, 1.6 mmol/L dNTPs, 1.5 mmol/L MgCl_2_, and 1 unit of *Taq* polymerase. The annealing temperature and the number of cycles required to observe the linear amplification range were determined for each assayed gene. Real-time RT-PCR was performed in an automated thermal cycler and detected with a Biorad detection system (Biorad, Munich, Germany), using SYBR Green fluorescence for quantification. The results were calculated using the 2^−ΔΔCt^ method, as described by Livak and Schmittgen [19]. Invitrogen (Karlsruhe, Germany) synthesized the primers used. Bestkeeper, which calculates a correlation with four housekeeping genes [18S RNA, hypoxanthine-guanine phosphoribosyltransferase (HPRT), glyceraldehyde 3-phosphate dehydrogenase (GAPDH), and beta-2-microglobulin (B2M)], was used as the internal control for mRNA expression [20]. The following primers were used for real-time RT-PCR experiments.

HPRT: forward 5′-CCA GCG TCG TGA TTA GTG AT-3′ 

HPRT: reverse 5′-CAA GTC TTT CAG TCC TGT CC-3′

B2M: forward 5′-GCC GTC GTG CTT GCC ATT C-3′

B2M: reverse 5′-CTG AGG TGG GTG GAA CTG AGA C-3′

GAPDH: forward 5′-TCC ATG CCA TCA CTG CCA CTC-3′

GAPDH: reverse 5′-TGA CCT TGC CCA CAG CCT TG-3′

ANT1: forward 5′-TAA GGA CTT CCT GGC AGG TG-3′

ANT1: reverse 5′-ACC CCT CCA GAA GGA GAG AA-3′

18s RNA: Qiagen, # QT00199374

### 2.5. Western Blot Analysis

Hearts were lysed in 1× cell lysis buffer (Cell Signaling, Beverly, MA, USA), containing 20 mmol/L Tris (pH 7.5), 150 mmol/L NaCl, 1 mmol/L EDTA, 1 mmol/L EGTA, 2.5 mmol/L sodium pyrophosphate, 1 mmol/L β-glycerophosphate, 1 mmol/L Na_3_VO_4_, 1% Triton X-100, and 1 µg/mL leupeptin, supplemented with 1× Complete Protease Inhibitor Cocktail (Roche, Basel, Switzerland) and subsequently centrifuged at 13,000× *g* for 10 min. The protein concentration of the supernatant was determined using the DC Protein Assay kit (BioRad, Hercules, CA, USA). Volumes containing 40 µg total protein were electrophoretically separated using 10% sodium dodecyl sulfate-polyacrylamide gel electrophoresis (SDS-PAGE) and transferred to nitrocellulose membranes [14]. For the analysis of PLB, the samples were not heated. Protein transfer was controlled by staining membranes with Ponceau S. Blots were blocked in 5% dry non-fat milk in Tris-buffered saline with 0.1% Tween 20 (TBST) at room temperature (RT) for 1 h. Western blots were performed using a standard protocol, with specific primary antibodies against total ANT (sc-9299, 1:1000), phospho-phospholamban (pPLB) (sc-12963, 1:1000), phospholamban (PLB), (Santa Cruz Biotechnology, Inc, Heidelberg, Germany), SERCA2a (S1314, 1:1000), ANT1 (SAB4300887, 1:1000) (Sigma-Aldrich, Taufkirchen, Germany), 4-Hydroxynonenal (HNE) (MBS169218, 1:8000) (My BioSource, Echingen, Germany), and caspase 3 (#9662, 1:1000), (Cell Signaling Technology, Frankfurt a. M., Germany). Antibody incubation was performed in blocking solution or 1% bovine serum albumin in TBST for the pPLB antibody overnight at 4 °C. Horseradish peroxidase (HRP)-conjugated goat anti-rabbit IgG or goat anti-mouse IgG was used as the secondary antibody (Cell Signaling Technology, Frankfurt a. M., Germany). Incubation was done in blocking solution for one hour at room temperature. Immuno-reactive bands were detected using SuperSignal West Femto Maximum Sensitivity Substrate (Pierce, Rockford, IL, USA). Protein bands were quantified with Quantity One software Version 4.6.9 (Bio-Rad Laboratories, USA). Ponceau S bands were used to normalize the protein expression levels of each blot [21].

### 2.6. Mitochondria Isolation

Mitochondria were isolated after normoxic (basal) Langendorff perfusion or after 45 min of ischemia plus two hours of reperfusion. All tubes and buffers were placed on ice. Whole heart tissue was first minced with scissors in ice-cold homogenization buffer A (100 mmol/L KCl, 50 mmol/L 3-[N-morpholino]-propane sulfonic acid (MOPS), 5 mmol/L MgSO_4_, 1 mmol/L EGTA, 1mM ATP, pH 7.4), and then homogenized six times with a 15 mL glass Potter. The homogenate was centrifuged at 800× *g* for 10 min at 4 °C. The supernatant containing mitochondria was removed and centrifuged at 8000× *g* for 10 min at 4 °C. The sedimented mitochondria were washed in homogenization buffer A and resuspended in a small volume of homogenization buffer A without ATP. Until measurements, the mitochondria remained on ice. 

### 2.7. Calcium Retention Assay

The calcium retention capacity (CRC) refers to the quantity of Ca^2+^ uptake by mitochondria prior to the MPTP opening. The CRC of 0.1 mg/mL mitochondria was measured in 2 mL incubation buffer (in mM: KCl 250, MOPS 20, KH_2_PO_4_ 0.0024, MgCl_2_ 0.0024, CaCl_2_ 0.0375, ADP 0.2) at 25 °C, using 5 mmol/L glutamate and 2.5 mmol/L malate as the substrates for complex I. Extramitochondrial Ca^2+^ was detected by 0.5 µmol/L calcium green 5 N (Invitrogen, Carlsbad, CA, USA) using a Cary Eclipse spectrophotometer at an excitation wavelength of 500 nm and an emission wavelength of 530 nm. Five mmol/L CaCl_2_ was added every three minutes until an increase in green calcium fluorescence was detected, which reflected the opening of the MPTP. These measurements were repeated in the presence of 1 µmol/L cyclosporine A, an inhibitor of the MPTP.

### 2.8. Membrane Potential (Δψ_m_)

To measure the membrane potential, 0.5 mg/mL mitochondria were incubated with 10 nmol/L 123 rhodamines, a lipophilic, cationic, membrane-permeable, and voltage-sensitive dye that distributes across the inner mitochondrial membrane Fluorescence was detected using a Cary Eclipse spectrophotometer (Varian, Mulgrave, Australia) at 25 °C, using 505 nm for excitation and 534 nm for emission. After 20 min, 40 µmol/L ADP was added, resulting in mitochondrial membrane potential depolarization. Changes in membrane potential were determined based on the observed increases in 123 rhodamine fluorescence after ADP addition [18] and shown as the percentages of the control (WT_basal_). 

### 2.9. Oxygen Consumption

The respiration of 0.1 mg/mL mitochondria was measured in a Clark-type oxygen electrode (Strathkelvin, Glasgow, UK) at 25 °C in incubation buffer (250 mmol/l KCl, 20 mmol/L MOPS, 2.4 mmol/L KH_2_PO_4_, 2.4 mmol/L MgCl_2_, 40 µmol/L EGTA, pH 7.4), using 5 mmol/L glutamate and 2.5 mmol/L malate as the substrates for complex I or 5 mmol/L succinate as the substrate for complex II, in the presence of 2 µM rotenone. Initially, basal oxygen consumption was recorded with continuous stirring of the mitochondrial suspension. After four minutes, 40 µmol/L ADP was added, and respiration was determined. Oxygen consumption was expressed in nmol O_2_·minute^−1^·mg protein^−1^ [22].

### 2.10. Statistical Analysis

Statistical analysis was performed using SPSS (IBM SPSS Statistics 26). The results are expressed as the mean ± standard error of the mean (SEM). All variables were evaluated for normal distribution using the Kolmogorov–Smirnov test. Levene’s test was used to control for variance homogeneity. A one-way analysis of variance (ANOVA), followed by the Sidak post hoc test, was used for variables with normal distribution and equal variance; the Kruskal–Wallis, followed by Dunn’s post hoc test, was used for variables with non-normal distribution and unequal variance; and the Mann–Whitney U test was applied for further evaluations of differences between two means. Values of *p* < 0.05 were considered significant.

## 3. Results

### 3.1. ANT Expression and Modification in I/R-Treated Hearts

**Figure 1:**
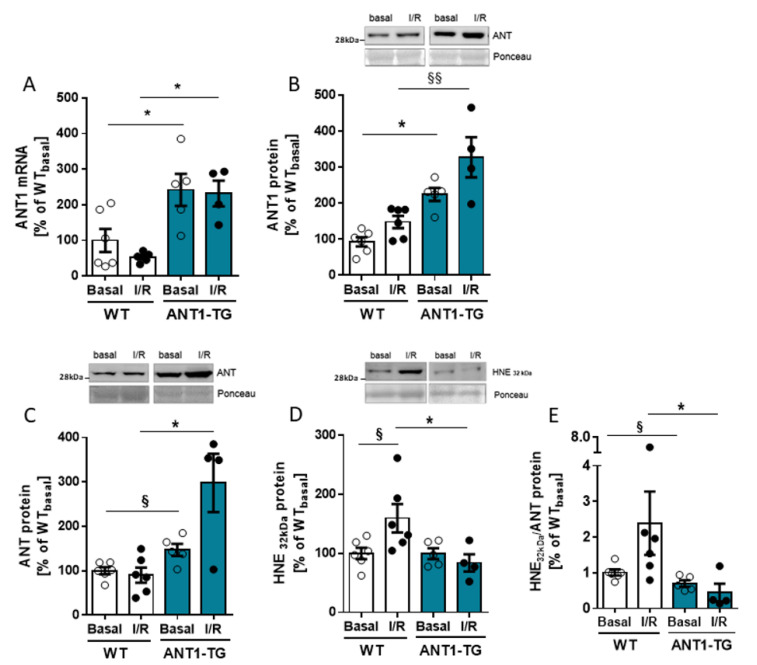
ANT expression and oxidative modification. Langendorff-perfused WT and ANT1-transgenic hearts were isolated after 15 min of basal perfusion (Basal) or after 45 min of ischemia, followed by two hours of reperfusion (I/R). Quantitative analysis of (**A**) ANT1 mRNA performed by real-time RT-PCR, (**B**) ANT1 protein, (**C**) total ANT protein, and (**D**) HNE-modified 32-kDa protein was done by Western blot analysis. The upper panels show representative Western blots, with Ponceau staining as the internal control. (**E**) The ratio between HNE_32kDa_/total ANT protein, indicating the proportion of HNE-modified ANT protein. Data are presented as the mean ± SEM, * *p* < 0.05 using one-way ANOVA or Kruskal–Wallis with respective post hoc tests or ^§^ *p* < 0.05, ^§§^ *p* < 0.01 using Mann–Whitney U test.

ANT1 mRNA and protein levels were significantly higher in ANT1-TG hearts than in WT hearts, under both basal conditions and after I/R (Figure 1A,B). The same was observed for the total ANT protein levels, including all ANT isoforms (Figure 1C). 4-Hydroxynonenal (HNE) is a lipid peroxidation product that modifies ANT protein and restricts ANT function [23]. A 32-kDa HNE-modified protein was previously identified as HNE-modified ANT protein [15]. The level of this HNE-modified protein increased in WT heart tissue but not in ANT1-TG heart tissue after I/R relative to the respective basal levels (Figure 1D). Compared with reperfused WT hearts, the HNE-modified ANT to total ANT protein ratio was significantly lower in reperfused ANT1-TG hearts (Figure 1E). Thus, ANT1 expression levels and the degree of oxidative modifications differed significantly between WT and ANT1-TG heart tissue under both normoxic conditions and after I/R.

### 3.2. ANT1 Overexpression Stabilizes Mitochondrial Calcium Retention Capacity and Membrane Potential after I/R

**Figure 2:**
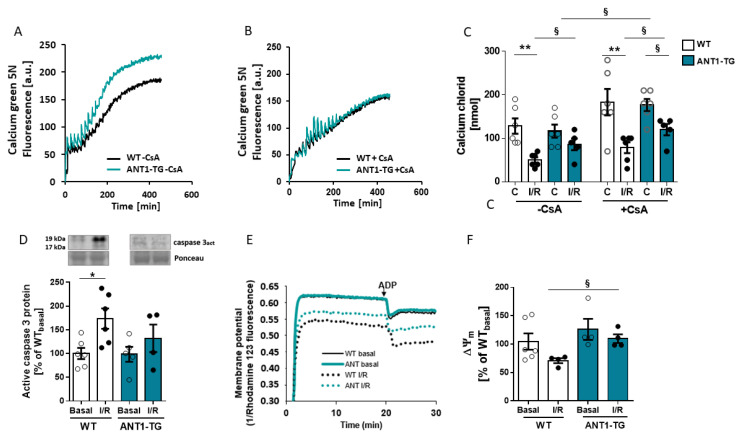
Calcium handling and membrane potential of cardiac mitochondria. Mitochondria were isolated from WT and ANT1-TG hearts after 15 min of basal perfusion (Basal) or after 45 min of ischemia, followed by two hours of reperfusion (I/R). Calcium retention capacities were measured in glutamate/malate buffer by adding 10 nmol CaCl_2_ to the mitochondria, in the presence or absence of cyclosporin A (CsA), every three minutes until the pore opened. Representative time courses are shown for WT and ANT1-TG mitochondria in the absence (**A**,**B**) presence of cyclosporin A. (**C**) The quantification of the calcium retention capacities for WT and ANT1-TG mitochondria, before and after I/R. (**D**) Quantitative analysis of active caspase 3 protein expression was performed by Western blot analysis. The upper panel shows representative Western blots, with Ponceau staining as the internal control. (**E**) The membrane potential was measured using rhodamine 123, and the differences in fluorescence emissions 1 min before and 1 min after the addition of ADP were measured. Increased dye emissions represent decreased membrane potential. (**F**) Quantification of the mitochondrial membrane potential (ΔΨ_m_). Data are presented as the mean ± SEM * *p* < 0.05, ** *p* < 0.01 using the Kruskal–Wallis test; or ^§^ *p* < 0.05 calculated with the Mann–Whitney U test.

Mitochondrial dysfunction is central to the pathogenesis of I/R injury, caused by a sudden increase in the permeability of the inner mitochondrial membrane due to the persistent opening of the MPTP, resulting in caspase activation. ANT1 is a regulator of the MPTP. The calcium-induced opening of the MPTP can be determined by the calcium retention capacity (CRC) of mitochondria. I/R treatment reduced the Ca^2+^ concentration required for MPTP opening in WT but not ANT1-TG mitochondria, seen by lower numbers of calcium uptakes during the measurement (Figure 2A–C). Cyclosporin A (CsA), an MPTP inhibitor, increased the Ca^2+^ concentration to open the MPTP in all groups but reached statistical significance only for normoxic ANT1-TG mitochondria. Even under CsA-mediated MPTP inhibition, the ANT1-TG mitochondria required higher calcium concentrations to initiate MPTP opening than WT mitochondria after I/R. This finding indicated that ANT1-TG are more resistant to calcium overload than WT mitochondria. The decrease in CRC in I/R WT mitochondria was linked to the increased expression of active caspase 3 protein in the heart tissue (Figure 2D).

MPTP opening significantly influences the membrane potential (ΔΨ_m_). Baseline ΔΨ_m_ values were similar between WT and ANT1-TG control mitochondria (Figure 2E,F). However, whereas ΔΨ_m_ decreased in WT mitochondria after I/R, no change was observed in ANT1-TG mitochondria (Figure 2F). 

### 3.3. ANT1 Overexpression Stabilizes Mitochondrial Oxygen Consumption after I/R 

**Figure 3:**
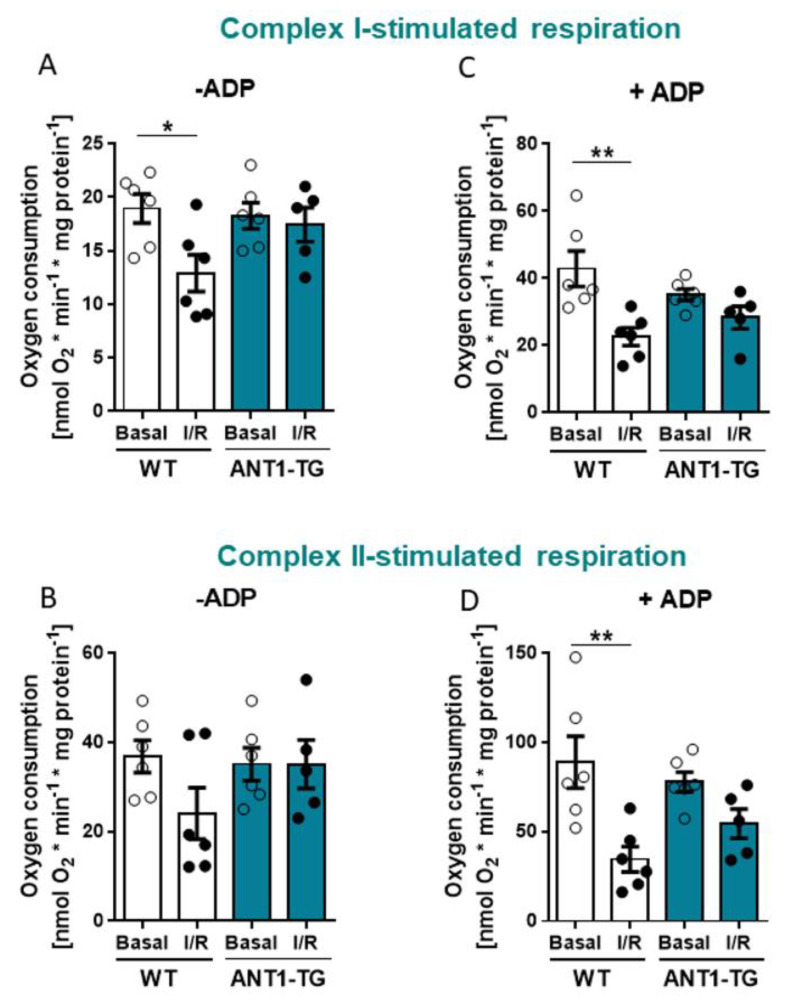
Mitochondrial respiration of cardiac mitochondria. WT and ANT1-TG mitochondria were isolated after 15 min of basal perfusion (Basal) or after 45 min of ischemia, followed by two hours of reperfusion (I/R), and the oxygen consumption was measured with and without the addition of 40 µmol/L ADP. Oxygen consumption in the presence of complex I substrates without ADP (**A**) or with ADP (**C**) and complex II substrates without ADP (**B**) or with ADP (**D**). Data represent the mean ± SEM, * *p* < 0.05, ** *p* < 0.01 using the one-way ANOVA or Kruskal–Wallis test with the respective post hoc tests.

Oxygen consumption by WT and ANT1-TG mitochondria were measured using complex I (glutamate/malate) and complex II substrates (succinate) (Figure 3). Mitochondria isolated from reperfused WT hearts showed reduced oxygen consumption compared to mitochondria without I/R treatment in the presence of the complex I substrate and the absence of ADP, whereas respiration in ANT1-TG mitochondria from reperfused hearts did not change (Figure 3A). In the presence of the complex II substrate without ADP, ANT1-TG showed again no reduction in respiration, and the oxygen consumption of WT mitochondria did not significantly decrease (Figure 3B). ADP-stimulated respiration (state 3) declined in WT mitochondria in the presence of the complex I substrate after I/R relative to mitochondria without I/R treatment. In contrast, no significant difference in ADP-stimulated respiration was observed in ANT1-TG mitochondria after I/R compared to mitochondria without I/R treatment when using the complex I substrate (Figure 3C). Similarly, in the presence of the complex II substrate ADP-stimulated respiration significantly decreased in WT mitochondria after reperfusion relative to mitochondria without I/R (Figure 3D). These results indicated that the respiratory function of ANT1-TG mitochondria was less harmed by I/R than that of WT mitochondria.

### 3.4. ANT1 Overexpression Reduces the PLB/SERCA2a Ratio and Stabilizes a Beneficial pPLB/PLB Ratio Leading to Reduced Diastolic Pressure after I/R

**Figure 4:**
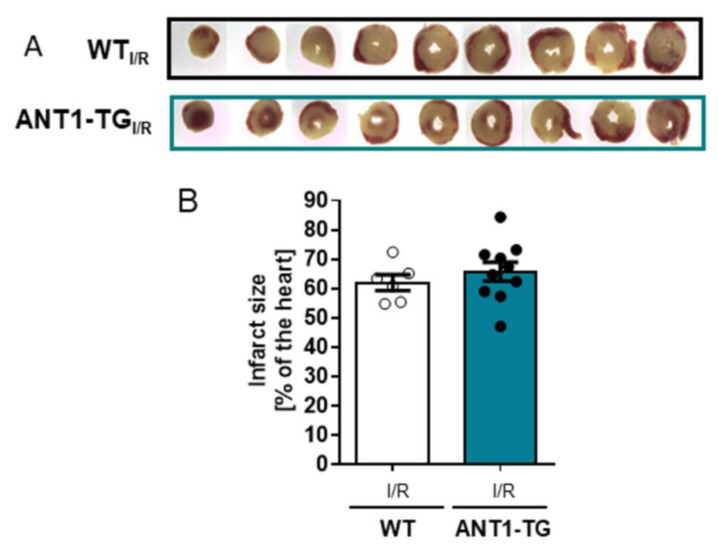
Determination of cell death in WT and ANT1-TG hearts after I/R (**A**,**B**) WT and ANT1-TG hearts were stained with TTC after I/R, and infarct sizes were measured as ratio to LV area (WT, *n* = 6; ANT1-TG, *n* = 10). (**A**) Representative images of one WT and one ANT1-TG heart, each with nine slices of the same heart. (**B**) Quantitative analysis of the infarct size after 45 min of ischemia, followed by two hours of reperfusion (I/R) from WT and ANT1-TG hearts. Data represent the mean ± SEM, * *p* < 0.05, using the one-way ANOVA.

Infarct sizes did not differ between WT and ANT1-TG hearts (Figure 4A,B). 

**Figure 5:**
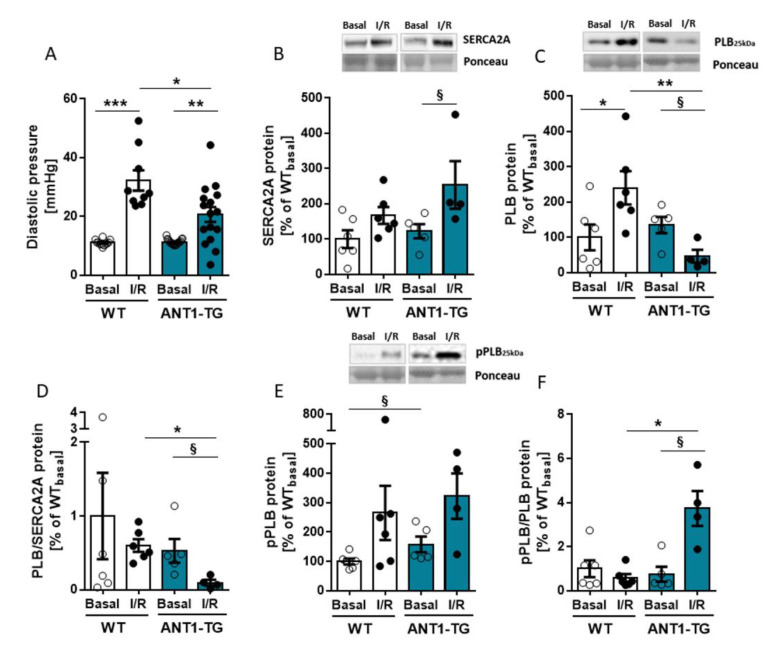
Diastolic pressure and calcium-handling proteins. (**A**) Diastolic pressure (P_dia_) of WT and ANT1-TG hearts. Data are shown for the pre-ischemic stabilization phase (Basal) and after 120 min of reperfusion (I/R) (WT, *n* = 10–12; ANT1-TG, *n* = 16–19). WT and ANT1-TG mitochondria were isolated after 15 min of basal perfusion (Basal) or after 45 min of ischemia, followed by two hours of reperfusion (I/R), and protein extracts were prepared. Figures show the (**B**) SERCA2a (**C**), PLB, (**D**) the PLB/SERCA2a ratio, (**E**) phosphorylated PLB (pPLB), and (**F**) the pPLB/PLB ratio, as assessed by Western blot quantification. Upper panels of graphs (**B**,**C**,**E**) show representative Western blots. Data are presented as the mean ± SEM from four to six heart preparations. Asterisks indicate significant differences * *p* < 0.05, ** *p* < 0.01, *** *p* < 0.001 using one-way ANOVA or Kruskal–Wallis with the respective post hoc tests or ^§^ *p* < 0.05 calculated using the Mann–Whitney U test.

However, the diastolic pressure increased less in the ANT1-TG than in WT hearts after I/R (Figure 5A), while other hemodynamic parameters were equal in WT and ANT1-TG hearts (Supplementary Table S1). 

The lower increase in diastolic pressure observed for ANT1-TG hearts following I/R may result from improved Ca^2+^ handling. Therefore, SERCA2a, one of the most important Ca^2+^ transporting proteins involved in muscle relaxation, and its regulator phospholamban (PLB) were analyzed. The protein expression levels of SERCA2a increased in ANT1-TG hearts after I/R (Figure 5B). The protein levels of PLB increased in WT hearts but decreased in ANT1-TG hearts after I/R (Figure 5C), resulting in a remarkable reduction in the PLB/SERCA2a ratio in ANT1-TG hearts after I/R, which was not observed in WT hearts (Figure 5D). The phosphorylation of PLB neutralizes its inhibitory effects on SERCA2a [24]. The ratio of phosphorylated to unphosphorylated PLB (pPLB/PLB) significantly increased in ANT1-TG hearts after I/R, whereas that in WT hearts remained unchanged after I/R (Figure 5E,F). Consequently, SERCA2a activity was less restricted by PLB regulation in ANT1-TG hearts than in WT hearts after I/R.

## 4. Discussion

Reperfused ANT1-TG Langendorff hearts displayed preserved mitochondrial function, as demonstrated by the maintenance of CRC and Δψ_m_ after I/R. In addition, the oxygen consumption of ANT1-overexpressing mitochondria was improved after I/R compared with that of WT mitochondria. ANT1-TG hearts demonstrated reduced diastolic dysfunction compared with WT hearts after I/R, and the smaller increase in the P_dia_ observed in ANT1-TG hearts relative to WT hearts was accompanied by a reduced PLB/SERCA2a ratio and diminished inhibitory PLB activity, which are both indicators of effective Ca^2+^ uptake into the SR.

ANT1-TG hearts expressed elevated ANT protein levels due to ANT1 overexpression. WT but not ANT1-TG hearts showed an increase in the oxidative HNE ANT modification under I/R conditions, and HNE modification has been shown to reduce ANT activity [23]. Thus, in contrast to WT hearts, ANT1-TG hearts were capable of reducing oxidative stress after I/R. Reduced oxidative ANT modification was associated with lower susceptibility to Ca^2+^ concentrations in ANT1-TG mitochondria after I/R, requiring significantly more Ca^2+^ to trigger MPTP opening. Reperfused ANT1-TG mitochondria also required higher Ca^2+^ concentrations than WT mitochondria to trigger MPTP opening in the presence of cyclosporin A, an MPTP inhibitor that binds to cyclophilin D (CypD). This finding suggests that the delayed MPTP opening may be independent of CypD in ANT1-TG mitochondria. Studies of new inhibitors support this possibility; C31, a strong inhibitor of MPTP opening, combines both CypD-dependent and CypD-independent inhibitory effects, although the exact mechanisms remain to be elucidated [25]. Moreover, Ca^2+^-induced MPTP opening was demonstrated in CypD KO mice, confirming the presence of a CypD-independent MPTP opening mechanism [26].

The improved CRC is consistent with the stable Δψ_m_ observed in ANT1-TG mitochondria after I/R, whereas a decrease in Δψ_m_ was observed in WT mitochondria after I/R. Thus, the increase in ANT protein supports the maintenance of Δψ_m_ under stressful conditions but does not increase Δψ_m_ beyond normal physiological levels under normoxic settings.

Complex I represents a major injury site within the respiratory chain during ischemia and is further damaged during reperfusion [27]. ANT is involved in one of the most important steps necessary for the regulation of oxidative phosphorylation and has been shown to control the steady-state respiration rates in mitochondria isolated from normal and ischemia-damaged hearts [28]. Our study demonstrated that ANT1 overexpression protects mitochondrial bioenergetics after I/R. Compared with WT mitochondria, steady-state basal mitochondrial respiration was not impaired in ANT1-TG mitochondria after I/R, as measured using both complex I and complex II substrates. ADP-stimulated respiration decreased in WT mitochondria but remained stable in ANT1-TG mitochondria after I/R. In addition, complex II substrate respiration decreased less in ANT1-TG mitochondria than in WT mitochondria, possibly due to increased complex II activity, which was previously reported in ANT1-TG hearts [12]. Thus, a higher level of unoxidized ANT protein led to a smaller decrease in oxygen consumption in ANT1-TG hearts compared with WT hearts. 

Mitochondrial dysfunction is a strong indicator of cell stress, activates caspase 3 and triggers cell death pathways. Caspase 3 is also necessary for chromatin condensation and DNA fragmentation [29]. The level of cleaved caspase 3 increased in total WT heart tissue after I/R relative to baseline but did not increase in ANT1-TG heart tissue after I/R. By comparison, the almost preserved mitochondrial function in ANT1-TG mitochondria did not prevent the loss of necrotic cardiac cells, which only represent the infarct zone. Nevertheless, the stable mitochondrial respiration and CRC observed in ANT1-TG hearts were accompanied by improved diastolic function. Thus, the surviving cardiomyocytes in the non-infarcted areas compensated for the infarct loss in ANT1-TG hearts, contributing to less restricted diastolic function after reperfusion. 

The preservation of low P_dia_ after I/R is another positive aspect of ANT1-TG hearts. Coronary blood flow occurs primarily during diastole [30]; thus, a good diastolic function is critical for maintaining the supply of oxygen and substrates to the heart. I/R results in diastolic dysfunction, as indicated by the increase in P_dia_. However, ANT1-TG hearts demonstrate reduced P_dia_ compared with WT hearts after I/R. During ischemia, the dissociation of myosin-actin cross-bridges becomes inhibited due to ATP depletion, initiating rigor contracture, which is a manifestation of Ca^2+^ overload and is associated with increased P_dia_ [31,32]. Additionally, rigor contracture activates myosin ATPase, consuming the remaining ATP [33] and inhibiting cross-bridge cycling, which represents the major ATP-dependent process in cardiomyocytes [34]. Reperfusion leads to pH level normalization, energy production recovery [35], and myofibril activation, which induces the development of a perpetual maximal force and results in hypercontracture [36]. Hypercontracture strength is correlated with the P_dia_ [4], which is caused by increased diastolic interactions among myofilaments, leading to intracellular Ca^2+^ overload [37]. Active Ca^2+^ transport into the SR is performed by SERCA2a [38] during heart relaxation. In our study, SERCA2a expression levels increased in ANT1-TG hearts after I/R. By contrast, PLB, the SERCA inhibitor, was upregulated in WT hearts but downregulated in ANT1-TG hearts, resulting in a significant decrease in the PLB/SERCA2a ratio in the ANT1-TG myocardium after I/R. This decreased ratio weakens the inhibitory function of PLB, facilitating SERCA2a activation and improving Ca^2+^ transport into the SR. Increased PLB/SERCA2a ratio results in heart failure due to reduced SR Ca^2+^ transport and impaired contractility [39]. Our previous studies revealed that untreated ANT1-TG hearts exhibited elevated SR Ca^2+^ transport due to increased SERCA2A activity [40]. Although we did not directly measure Ca^2+^ in the SR or cytosol in this study, the increase in the PLB/SERCA2a ratio suggested increased SR Ca^2+^ transport in reperfused ANT1-TG hearts. In addition, the pPLB/PLB ratio remained unchanged in WT heart tissue but was significantly upregulated in ANT1-TG hearts following I/R, which might be due to the preserved mitochondrial function and the associated maintenance of ATP production capacity. Dephosphorylated PLB is a SERCA inhibitor, whereas PLB phosphorylation leads to SERCA2a activation [24], contributing to the observed decrease in cytosolic Ca^2+^ during relaxation [41]. These findings correspond to our observation that ANT1-TG mitochondria were more resistant to Ca^2+^ concentration changes than WT mitochondria. Therefore, ANT1 overexpression supports improved Ca^2+^ handling in the heart, which positively affects relaxation during I/R.

We previously described the cardioprotective effect of ANT1 overexpression on ischemic hearts in vivo [15]. In contrast, in our current in vitro setting, ANT1 overexpression preserved the mitochondrial function but did not affect the infarct size after I/R. The methodological approach was 45 min of ischemia with 2 h of reperfusion and a normoxic control of 15 min, equivalent to the pre-ischemic time point. A normoxia time of three hours would consider the ex vivo time of the heart, but the perfusion of the heart produces shear stress over the entire period, which is absent during ischemia. The issue of appropriate normoxia control was already a topic addressed by Heidorn et al. (2018). Experiments were performed using different normoxia time points taken over 180 min. As a result, no differences were observed in the hemodynamics at any of the measured time intervals during normoxia. These facts justify our experimental design. The differences between our present study and the outcome in the in vivo study (15) are the following: (1). The study described here analyzed myocardial I/R, unlike the previous study, which investigated permanent ischemia after 24 h. (2). In the present study, we used the Langendorff technique as an in vitro model, which enabled the influence of ANT1 overexpression to be assessed in the heart independently of the effects on the entire organism. Hence remodeling processes initiated by the organism or intercellular communications within the heart may have additional effects in ANT1-TG animals. We found that heart-protecting factors, such as the increased expression of the heat shock protein 27 (HSP27), were down-regulated in ANT1-TG hearts during I/R (Supplementary Figure S1), whereas HSP27 remained stable in ischemic hearts [14]. Because HSP27 has several extracellular and intracellular functions in modulating cell signaling and vascular and immunological processes [42], the loss of HSP27, which also displays pronounced anti-apoptotic and anti-oxidative properties, reduce the cardioprotective effect of ANT1 overexpression and may explain similar infarct sizes observed in WT and ANT1-TG hearts. But even with unchanged infarct size, the remaining undamaged cells better compensate for cell loss in reperfused ANT1-TG than in WT hearts. The findings of Wang et al. [43] support the theory that intercellular communication is necessary to protect ANT1-TG hearts. They demonstrated that the adenoviral overexpression of ANT1 inhibits H_2_O_2_-induced necrotic cell death in neonatal cardiomyocytes, and I/R-induced myocardial necrosis in vivo is reflected in the infarct size. Thus, the infarct size may well have been smaller in an in vivo model of I/R in ANT1-overexpressing rats rather than the Langendorff model used here. In addition, improvement in mitochondrial function does not always translate into a reduction in infarct size. This was shown by M. Szibor et al. [44]. The respiratory enzyme AOX (alternative oxidase) preserved the electron flux of stressed mitochondria and attenuated ROS production but had no benefit for the infarction size of the heart during the acute phase of I/R (30′ ischemia/120′ reperfusion) in AOX overexpressing mice. The protection only occurred later during remodeling; that may also be the case in the reperfused ANT1-TG hearts. 

ANT1 overexpression alters molecular and physiological processes, some of which are also associated with cardiac preconditioning. The ANT content declines in non-preconditioned hearts during reperfusion, whereas the ANT level in preconditioned hearts remains stable during I/R [45]. Preconditioning also preserves the mitochondrial oxygen consumption rate [46]. Delayed ischemic preconditioning, during which ANT1 was upregulated, was found to alter mitochondrial function by improving the energy balance and the efficiency of oxygen consumption [47]. In addition, Wang et al. [43] used an in vivo mouse model to demonstrate that the knockdown of miR-2861 increases ANT1 expression, which had a positive effect on I/R hearts. Although these studies indirectly link ANT1 to beneficial effects for the heart, we demonstrated that increased ANT1 expression was directly responsible for protective cardiac processes during I/R. Thus, stable ANT1 expression, induced through preconditioning or genetic manipulation, is advantageous for the heart and may serve as a promising therapeutic target during reperfusion to support cardiac function.

## Data Availability

The datasets generated for this study are available upon request to the corresponding author.

## Supplementary Materials

The following are available online at https://www.mdpi.com/article/10.3390/membranes11110836/s1, Figure S1: HSP27 expression of rat hearts after I/R, Table S1: The following hemodynamic parameters were recorded during the pre-ischemic stabilization phase (baseline) and after 120 min (R 120) of reperfusion: the maximal rate of left ventricular pressure (dP/dt max), the minimal rate of left ventricular pressure (dP/dt min), systolic pressure (P sys), (left ventricular developed pressures (LVDPs) and heart rates (HRs). WT, *n* = 15, ANT1-TG, *n* = 19. Data represent the mean ± SEM., *p* < 0.05; *, *p* < 0.01; ***, *p* < 0.001 for baseline vs. reperfusion with unpaired *t*-test.

## References

[1] Halestrap A.P., Connern C.P., Griffiths E.J., Kerr P.M. (1997). Cyclosporin A binding to mitochondrial cyclophilin inhibits the permeability transition pore and protects hearts from ischaemia/reperfusion injury. Detect. Mitochondrial Dis..

[2] Valverde C.A., Kornyeyev D., Ferreiro M., Petrosky A.D., Mattiazzi A., Escobar A.L. (2010). Transient Ca^2+^ depletion of the sarcoplasmic reticulum at the onset of reperfusion. Cardiovasc. Res..

[3] Hausenloy D.J., Yellon D.M. (2013). Myocardial ischemia-reperfusion injury: A neglected therapeutic target. J. Clin. Investig..

[4] Ladilov Y., Efe O., Schäfer C., Rother B., Kasseckert S., Abdallah Y., Meuter K., Schlüter K.D., Piper H.M. (2003). Reoxygenation-induced rigor-type contracture. J. Mol. Cell. Cardiol..

[5] Iwanaga Y., Hoshijima M., Gu Y., Iwatate M., Dieterle T., Ikeda Y., Date M.O., Chrast J., Matsuzaki M., Peterson K.L. (2004). Chronic phospholamban inhibition prevents progressive cardiac dysfunction and pathological remodeling after infarction in rats. J. Clin. Investig..

[6] Klingenberg M. (2008). The ADP and ATP transport in mitochondria and its carrier. Biochim. Biophys. Acta.

[7] Ruprecht J.J., Kunji E.R.S. (2020). The SLC25 Mitochondrial Carrier Family: Structure and Mechanism. Trends Biochem. Sci..

[8] Carraro M., Carrer A., Urbani A., Bernardi P. (2020). Molecular nature and regulation of the mitochondrial permeability transition pore(s), drug target(s) in cardioprotection. J. Mol. Cell. Cardiol..

[9] Wang X., Salinas K., Zuo X., Kucejova B., Chen X.J. (2008). Dominant membrane uncoupling by mutant adenine nucleotide translocase in mitochondrial diseases. Hum. Mol. Genet..

[10] Liu Y., Wang X., Chen X.J. (2015). Misfolding of mutant adenine nucleotide translocase in yeast supports a novel mechanism of Ant1-induced muscle diseases. Mol. Biol. Cell.

[11] Dörner A., Schultheiss H.P. (2007). Adenine Nucleotide Translocase in the Focus of Cardiovascular Diseases. Trends Cardiovasc. Med..

[12] Walther T., Tschöpe C., Sterner-Kock A., Westermann D., Heringer-Walther S., Riad A., Nikolic A., Wang Y., Ebermann L., Siems W.E. (2007). Accelerated mitochondrial adenosine diphosphate/adenosine triphosphate transport improves hypertension-induced heart disease. Circulation.

[13] Heger J., Abdallah Y., Shahzad T., Klumpe I., Piper H.M., Schultheiss H.P., Schlüter K.D., Schulz R., Euler G., Dörner A. (2012). Transgenic overexpression of the adenine nucleotide translocase 1 protects cardiomyocytes against TGFbeta(1)-induced apoptosis by stabilization of the mitochondrial permeability transition pore. J. Mol. Cell. Cardiol..

[14] Winter J., Hammer E., Heger J., Schultheiss H.P., Rauch U., Landmesser U., Dörner A. (2019). Adenine Nucleotide Translocase 1 Expression is Coupled to the HSP27-Mediated TLR4 Signaling in Cardiomyocytes. Cells.

[15] Klumpe I., Savvatis K., Westermann D., Tschöpe C., Rauch U., Landmesser U., Schultheiss H.P., Dörner A. (2016). Transgenic overexpression of adenine nucleotide translocase 1 protects ischemic hearts against oxidative stress. J. Mol. Med..

[16] Heidorn M., Frodermann T., Böning A., Schreckenberg R., Schlüter K.D. (2018). Citrulline Improves Early Post-Ischemic Recovery or Rat Hearts In Vitro by Shifting Arginine Metabolism from Polyamine to Nitric Oxide Formation. Clin. Med. Insights Cardiol..

[17] Maier T., Schreckenberg R., Schlüter K.D. (2009). Effect of preischemic beta-adrenoceptor stimulation on postischemic contractile dysfunction. Life Sci..

[18] Boengler K., Bulic M., Schreckenberg R., Schlüter K.D., Schulz R. (2017). The gap junction modifier ZP1609 decreases cardiomyocyte hypercontracture following ischaemia/reperfusion independent from mitochondrial connexin 43. Br. J. Pharmacol..

[19] Livak K.J., Schmittgen T.D. (2001). Analysis of relative gene expression data using real-time quantitative PCR and the 2(-Delta Delta C(T)) Method. Methods.

[20] Pfaffl M.W., Tichopad A., Prgomet C., Neuvians T.P. (2004). Determination of stable housekeeping genes, differentially regulated target genes and sample integrity: BestKeeper—Excel-based tool using pair-wise correlations. Biotechnol. Lett..

[21] Boengler K., Bencsik P., Palóczi J., Kiss K., Pipicz M., Pipis J., Ferdinandy P., Schlüter K.D., Schulz R.L. (2017). Lack of Contribution of p66shc and Its Mitochondrial Translocation to Ischemia-Reperfusion Injury and Cardioprotection by Ischemic Preconditioning. Front. Physiol..

[22] Hirschhäuser C., Bornbaum J., Reis A., Böhme S., Kaludercic N., Menabò R., Di Lisa F., Boengler K., Shah A.M., Schulz R. (2015). NOX4 in Mitochondria: Yeast Two-Hybrid-Based Interaction with Complex I Without Relevance for Basal Reactive Oxygen Species?. Antioxid. Redox Signal..

[23] Yan L.J., Sohal R.S. (1998). Mitochondrial adenine nucleotide translocase is modified oxidatively during aging. Proc. Natl. Acad. Sci. USA.

[24] Kranias E.G., Hajjar R.J. (2012). Modulation of cardiac contractility by the phospholamban/SERCA2a regulatome. Circ. Res..

[25] Panel M., Ahmed-Belkacem A., Ruiz I., Guichou J.F., Pawlotsky J.M., Ghaleh B., Morin D.A. (2021). Phenyl-Pyrrolidine Derivative Reveals a Dual Inhibition Mechanism of Myocardial Mitochondrial Permeability Transition Pore, Which Is Limited by Its Myocardial Distribution. J. Pharmacol. Exp. Ther..

[26] Baines C.P., Kaiser R.A., Purcell N.H., Blair N.S., Osinska H., Hambleton M.A., Brunskill E.W., Sayen M.R., Gottlieb R.A., Dorn G.W. (2005). Loss of cyclophilin D reveals a critical role for mitochondrial permeability transition in cell death. Nature.

[27] Solaini G., Harris D.A. (2005). Biochemical dysfunction in heart mitochondria exposed to ischaemia and reperfusion. Biochem. J..

[28] Borutaite V., Mildaziene V., Katiliutt Z., Kholodenko B., Toleikis A. (1993). The function of ATP/ADP translocator in the regulation of mitochondrial respiration during development of heart ischemic injury. Biochim. Biophys. Acta.

[29] Porter A.G., Jänicke R.U. (1999). Emerging roles of caspase-3 in apoptosis. Cell Death Differ..

[30] Heusch G. (2008). Heart rate in the pathophysiology of coronary blood flow and myocardial ischaemia: Benefit from selective bradycardic agents. Br. J. Pharmacol..

[31] Garcia-Dorado D., Ruiz-Meana M., Inserte J., Rodriguez-Sinovas A., Piper H.M. (2012). Calcium-mediated cell death during myocardial reperfusion. Cardiovasc. Res..

[32] Gutierrez C., Blanchard D. (2004). Diastolic Heart Failure: Challenges of Diagnosis and Treatment. Am. Fam. Physician.

[33] Bowers K.C., Allshire A.P., Cobbold P.H. (1992). Bioluminescent measurement in single cardiomyocytes of sudden cytosolic ATP depletion coincident with rigor. J. Mol. Cell. Cardiol..

[34] Tran K., Loiselle D.S., Crampin E.J. (2015). Regulation of cardiac cellular bioenergetics: Mechanisms and consequences. Physiol. Rep..

[35] Piper H.M., Garcia-Dorado D., Ovize M. (1998). A fresh look at reperfusion injury. Cardiovasc. Res..

[36] Siegmund B., Schlüter K.D., Piper H.M. (1993). Calcium and the oxygen paradox. Cardiovasc. Res..

[37] Calderón-Sánchez E.M., Domínguez-Rodríguez A., López-Haldón J., Jiménez-Navarro M.F., Gómez A.M., Smani T., Ordóñez A. (2016). Cardioprotective Effect of Ranolazine in the Process of Ischemia-reperfusion in Adult Rat Cardiomyocytes. Revista Española de Cardiología.

[38] Dibb K.M., Graham H.K., Venetucci L.A., Eisner D.A., Trafford A.W. (2007). Analysis of cellular calcium fluxes in cardiac muscle to understand calcium homeostasis in the heart. Cell Calcium.

[39] Antoons G. (2003). Regulation of Ca^2+^ Release from the Sarcoplasmic Reticulum in the Normal and Failing Heart.

[40] Vogelpohl I., Vetter R., Heger J., Ebermann L., Euler G., Schultheiss H.P., Dörner A. (2011). Transgenic overexpression of heart-specific adenine nucleotide translocase 1 positively affects contractile function in cardiomyocytes. Cell. Physiol. Biochem..

[41] Vittone L., Mundina-Weilenmann C., Mattiazzi A. (2008). Phospholamban phosphorylation by CaMKII under pathophysiological conditions. Front. Biosci..

[42] Batulan Z., Pulakazhi Venu V.K., Li Y., Koumbadinga G., Alvarez-Olmedo D.G., Shi C., O’Brien E.R. (2016). Extracellular Release and Signaling by Heat Shock Protein 27: Role in Modifying Vascular Inflammation. Front. Immunol..

[43] Wang K., Long B., Li N., Li L., Liu C.Y., Dong Y.H., Gao J.N., Zhou L.Y., Wang C.Q., Li P.F. (2016). MicroRNA-2861 regulates programmed necrosis in cardiomyocyte by impairing adenine nucleotide translocase 1 expression. Free Radic. Biol. Med..

[44] Szibor M., Schreckenberg R., Gizatullina Z., Dufour E., Wiesnet M., Dhandapani P.K., Debska-Vielhaber G., Heidler J., Wittig I., Nyman T.A. (2020). Respiratory chain signalling is essential for adaptive remodelling following cardiac ischaemia. J. Cell. Mol. Med..

[45] Yabe K., Nasa Y., Sato M., Lijima R., Takeo S. (1997). Preconditioning preserves mitochondrial function and glycolytic flux during an early period of reperfusion in perfused rat hearts. Cardiovasc. Res..

[46] Takeo S., Nasa Y. (1999). Role of energy metabolism in the preconditioned heart—A possible contribution of mitochondria. Cardiovasc. Res..

[47] McLeod C.J., Jeyabalan A.P., Minners J.O., Clevenger R., Hoyt R.F., Sack M.N. (2004). Delayed ischemic preconditioning activates nuclear-encoded electron-transfer-chain gene expression in parallel with enhanced postanoxic mitochondrial respiratory recovery. Circulation.

