# Supplementary material for: Mitochondria Isolated from Hearts Subjected to Ischemia/Reperfusion Benefit from Adenine Nucleotide Translocase 1 Overexpression
Source: Membranes (Basel). 2021 Oct 29;11(11):836. doi: 10.3390/membranes11110836 (PMC8619488; doi:10.3390/membranes11110836)
Supplement: Supplementary file 1 [file membranes-11-00836-s001.zip › membranes-1282710-supplementary.pdf]

Article

Mitochondria isolated from hearts subjected to ischemia/reperfusion benefit from adenine nucleotide translocase 1 overexpression

Andrea Dörner[1,2], Oleg Lynetskiy[3], Gerhild Euler[3], Ulf Landmesser[1,2], Klaus-Dieter Schlüter[3] and Jacqueline Heger[3]*

1 Charité – Universitätsmedizin Berlin, corporate member of Freie Universität Berlin, Humboldt-Universität zu Berlin, Institute of Health, Department of Cardiology, Centrum 11, Campus Benjamin Franklin, Berlin, Germany; andrea.doerner@charite.de (A.D.); Ulf.Landmesser@charite.de (U.L.)

2 DZHK (German Centre for Cardiovascular Research, partner site Berlin, Berlin, Germany)

3 Justus-Liebig-Universität Gießen, Physiologisches Institut, Gießen, Germany; Oleg-Lynetskiy@gmx.de (O.L.); Gerhild.Euler@physiologie.med.uni-giessen.de (G.E.); Klaus-Dieter.Schlueter@physiologie.med.uni-giessen.de (K-D.S.); Jacqueline.Heger@physiologie.med.uni-giessen.de (J.H.)

* Jacqueline.Heger@physiologie.med.uni-giessen.de

Hemodynamic parameters

	dP/dt max (mmHg/sec)	
	Baseline	R120
WT	2600 ± 171	1150 ± 148***
ANT1-TG	2237 ± 108	1316 ± 115***
	dP/dt min (mmHg/sec)	
	Baseline	R120
WT	-1513 ± 119	-836 ± 93***
ANT1-TG	-1384 ± 85	-889 ± 72***
	P sys (mmHg)	
	Baseline	R120
WT	87.9 ± 9.3	62.4 ± 6.7*
ANT1-TG	73.4 ± 3.1	57.9 ± 4.3**
	LVDP (mmHg)	
	Baseline	R120
WT	78.1 ± 9.6	30.3 ± 4.3***
ANT1-TG	62.0 ± 3.2	34.3 ± 3.1***
	HR (bpm)	
	Baseline	R120
WT	206.5 ± 15.0	231.2 ± 24.3
ANT1-TG	212.7 ± 8.8	239.9 ± 14.0

Table S1. The following hemodynamic parameters were recorded during the pre-ischemic stabilization phase (baseline) and after 120 minutes (R 120) of reperfusion: the maximal rate of left ventricular pressure (dP/dt max), the minimal rate of left ventricular pressure (dP/dt min), systolic pressure (P sys), (left ventricular developed pressures (LVDPs) and heart rates (HRs). WT, n=15, ANT1-TG, n=19. Data represent the mean ± SEM. *, p <0.05; **, p <0.01; ***, p<0.001 for baseline vs reperfusion with unpaired t-test.

Western blot analysis

Western blots were performed using a standard protocol (As described in main text), with specific primary antibodies against HSP27 (Santa Cruz Biotechnology, Inc, Heidelberg, Germany).

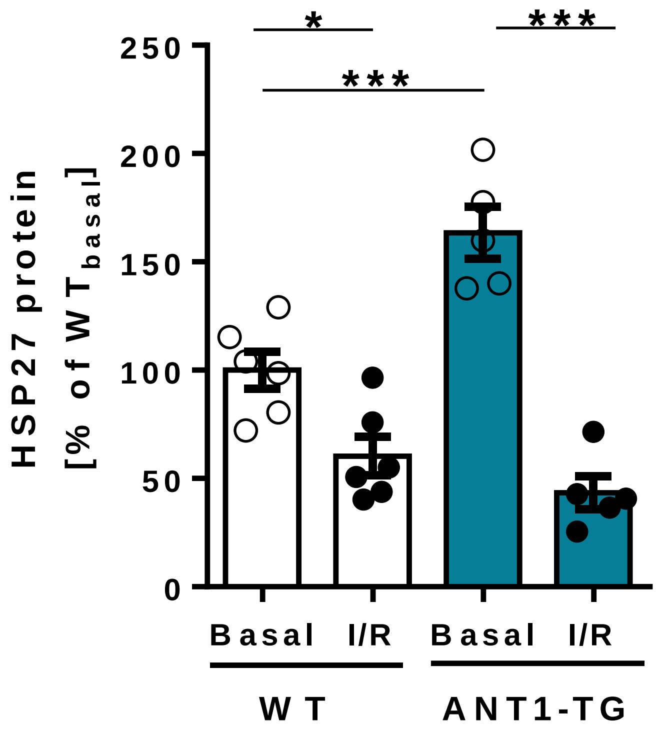

Figure S1. HSP27 expression of rat hearts after I/R. Langendorff-perfused WT and ANT1-transgenic hearts were exposed to 45 minutes of ischemia, followed by two hours of reperfusion. Graph shows the quantitative analysis of HSP27 protein. Data are shown for the pre-ischemic stabilization phase (basal) and after 120 minutes of reperfusion (I/R). Data are presented as the mean ± SEM, *, $p < 0.05$, **, $p < 0.01$, ***, $p < 0.001$ determined by ANOVA.
